# Clinicopathological evaluation of triple-negative breast cancer treated with keynote-522 regimen

**DOI:** 10.1093/oncolo/oyaf231

**Published:** 2025-07-24

**Authors:** Thaer Khoury, Shipra Gandhi, Gary Tozbikian, Oluwole Fadare, Samira Syed, Briana To, Dionisia Quiroga, Song Yao, Kristopher Attwood, Han Yu, Yisheng Fang

**Affiliations:** Department of Pathology, Roswell Park Comprehensive Cancer Center, Buffalo, NY, United States; Department of Oncology, Roswell Park Comprehensive Cancer Center, Buffalo, NY, United States; Department of Pathology, Wexner Medical Center at The Ohio State University, Columbus, OH, United States; Department of Pathology, Division of Anatomic Pathology, University of California San Diego Health, San Diego, CA, United States; Division of Hematology Oncology, University of Texas Southwestern Medical Center, Dallas, TX, United States; Pelotonia Institute for Immuno-Oncology, The James Comprehensive Cancer Center, The Ohio State University, Columbus, OH, United States; Pelotonia Institute for Immuno-Oncology, The James Comprehensive Cancer Center, The Ohio State University, Columbus, OH, United States; Department of Cancer Prevention and Control, Roswell Park Comprehensive Cancer Center, Buffalo, NY, United States; Department of Biostatistics, Roswell Park Comprehensive Cancer Center, Buffalo, NY, United States; Department of Biostatistics, Roswell Park Comprehensive Cancer Center, Buffalo, NY, United States; Division of Pathology Oncology, University of Texas Southwestern Medical Center, Dallas, TX, United States

**Keywords:** triple-negative breast cancer, immune checkpoint inhibitor, keynote-522, personalized medicine, de-escalation

## Abstract

**Background:**

Our objective is to compare the response rate between two matched cohorts of early-stage TNBC (chemotherapy: CT + immune checkpoint inhibitor: ICI vs CT alone) and to define clinicopathological variables to identify a subgroup of patients who could be spared or benefit from ICI.

**Methods:**

Patients treated with CT + ICI according to KEYNOTE-522 (KN-522) (*n* = 128) were included in the study and matched 1:1 with patients treated with CT alone. Matching criteria included age range (10 years), race, clinical stage (c)-AJCC, and histological type (metaplastic [MpBC] vs no special type [IC-NST]). The following histological characteristics in the core needle biopsy (CNB) were included: histological type, Nottingham grade, degree of necrosis, and percentage of tumor-infiltrating lymphocytes (TILs). The residual cancer burden (RCB) was categorized into 0 (pCR), I, II, or III. To identify a subgroup of patients who could be spared or benefit from adding ICI, an analysis of the penalized maximum likelihood estimate was performed.

**Results:**

pCR was achieved in 50% of patients treated with CT + ICI vs 35.9% treated with CT alone (*P* = .02). In the CT group, lower TILs in CNB, non-Black race, MpBC histology, and a higher degree of necrosis were associated with non-pCR. Patients were categorized into quartiles (Q) based on the predicted risk of non-pCR to CT (Q1 represents the lowest risk and Q4 represents the highest risk of non-pCR). In Q4, the pCR rate with CT alone was only 2.9% (1/46) vs 20% (6/64) in the CT + IT cohort (*P* = .044). The following variables were associated with Q4 vs Q1-3: non-Black race (96.9% vs 88%, *P* = .04), MpBC histology (29.7% vs 1.6%, *P* < .01), lymph node positive disease (54.6% vs 50%, *P* < .01), Nottingham grade 2 (32.8% vs 6.3%, *P* < .01), and lower mean TILs (12.8 ± 15 vs 36.7 ± 26.5, *P* < .01).

**Conclusions:**

The efficacy of CT + ICI regimen in the real world, although higher than CT, is lower than that observed on the KN 522 clinical trial. Our results need validation in a larger cohort.

Implications for Practice Immunotherapy (IT) with checkpoint inhibition along with chemotherapy (CT) were accepted to treat patients with triple-negative breast cancer (TNBC) in the neoadjuvant (NA) setting (KEYNOTE-522). Pembrolizumab is associated with several immune-related toxicities. With NACT alone, around 40% of patients achieve pathologic complete response (pCR). Therefore, there is a serious concern that we are potentially overtreating many of our patients in this setting, who can be cured just with CT alone. We aimed to define clinicopathologic variables to specify certain patients who may benefit from adding IT. We found these clinical and pathological variables associated with better response to ICI +CT, non-Black race, metaplastic carcinoma histology, lymph node positive, Nottingham grade 2, and lower tumor-infiltrating lymphocytes.

## Introduction

Triple-negative breast cancer (TNBC) subtype is a heterogeneous and the most aggressive form of BC with significantly shorter overall survival.^1^^,^^2^ It is defined by negative expression of the estrogen receptor (ER), progesterone receptor (PR), and HER2.^3^^,^^4^ KEYNOTE-522 (KN-522) established immune checkpoint inhibitor (ICI)—pembrolizumab addition to platinum-based chemotherapy (CT)—as the new standard of care for early-stage TNBC. KN-522 trialists randomized patients with untreated c-AJCC-stage 2/3 TNBC in a 2:1 ratio to pembrolizumab with carboplatin-paclitaxel followed by doxorubicin-cyclophosphamide or placebo with carboplatin-paclitaxel combination followed by doxorubicin-cyclophosphamide before surgery. The trial showed that patients treated with pembrolizumab with chemotherapy had a higher pathologic complete response (pCR): 64.8%, event-free survival (EFS) (81.2%), and overall survival (OS) (86.6%) at 5 years than those treated with CT alone: pCR: 51.2%, EFS 72.2%, and OS 81.7%. They also reported immune-related adverse events (irAEs), such as cardiomyositis, type 1 diabetes mellitus, and adrenal insufficiency, that can be life-threatening and permanent.^5^ Since 51.2% can achieve pCR with CT alone, it is likely that these patients are being subjected to potentially life-threatening immune-related adverse events (irAE) with CT + ICI combination, as only 13.6% (64.8%-51.2%) patients seem to derive the benefit from the addition of ICI. In addition, there is also a subset of patients that could achieve a decrease in residual cancer burden (RCB) by adding ICI to CT.^6^

Several factors may play a role in the response to neoadjuvant treatment (NAT) with CT + ICI or CT alone, including age, race, menopausal status, *BRCA1/2* mutation, tumor characteristics such as tumor grade, Ki-67 proliferation index, clinical stage, tumor mutational burden (TMB), tumor-infiltrating lymphocytes (TILs), and expression of programmed death ligand 1 (PD-L1).^7-11^ Therefore, there is an opportunity to identify clinicopathological variables that are associated with preferential benefit with the addition of ICI to CT.

The purpose of this study is, first, to evaluate the response rate of the CT + ICI regimen in the real world in patients with early-stage TNBC; second, to match these with cases treated with CT alone and to examine the clinical and pathological variables associated with the response to CT alone or CT + ICI; and third, to identify variables that would predict benefit with CT alone or additional benefit with ICI to CT.

## Materials and methods

### Patients (CT ± ICI group)

The cases were identified in four academic institutions (Roswell Park Comprehensive Cancer Center [RPCCC], Buffalo NY; Ohio State University [OHS], Columbus OH; University of Texas [UT] Southwestern, Dallas TX; and University of California at San Diego [UCSD], San Diego, CA). After obtaining approval from the Institutional Review Boards (IRB), the database was searched in each of the corresponding institutions for eligible patients between September 2021 and December 2022. First, patients with TNBC treated with CT + ICI were identified. Then, potential cases diagnosed and treated between April 2000 and September 2021 eligible for matching (treated with CT alone) were identified.

### Histological interpretation

The hematoxylin and eosin (H&E) slides of core needle biopsies (CNB) and excisional biopsies (EB) of both groups (treated with CT alone and CT + ICI) were interpreted by a specialized breast pathologist in each respective institution, with each contributor having more than 10 years of experience. When there was a question about how to interpret a variable, a representative image of the questionable area/variable was centrally reviewed.

### Histological interpretation of the CNB

The H&E slides were reviewed, and the following variables were recorded, including histological type, Nottingham grade (NG), percentage of TILs, and degree of necrosis. The histological subtype was classified according to the World Health Organization classification, including invasive carcinoma of no special type (IC-NST) and special types (in this study only two were recorded: metaplastic [MpBC] and apocrine).^12^ The tumor was graded following the NG system, in which tubular formation, nuclear pleomorphism, and mitotic count were incorporated, resulting in a score ranging from 1 to 3.^13^ The percentage of TILs was recorded that ranged from 0% to 100% following the 2014 International TILs Working Group.^14^ The percentage was calculated by dividing the area occupied by mononuclear inflammatory cells by the total stromal area multiplied by 100. TILs were evaluated only in the vicinity of the tumor and the intervening areas, and excluding the areas surrounding normal terminal ductal lobular units and ductal carcinoma in situ (DCIS).^15^ The degree of necrosis was graded on a scale from 0 (no necrosis) to 4 (geographic necrosis), where grade 1 = single focus (a cluster of >3 cells), grade 2 = two or more non-connected foci, grade 3 = two or more connected foci, and grade 4 = geographic necrosis.^16^

A tumor was considered triple negative when ER, PR, and HER2 were negative. ER/PR were considered negative when the staining was <1% of the tumor cells. HER2 was considered negative when the staining was 0+, 1+, or 2+ by immunohistochemistry and negative by in situ hybridization. The results of the biomarkers were abstracted from the pathology reports. For the CT + ICI group, the interpretation of the biomarkers followed the latest guidelines^3^^,^^4^; for the CT group, the guidelines at the time of diagnosis were followed.^3^^,^^4^^,^^17^^,^^18^

### Histological evaluation of the excisional biopsy

To calculate the RCB, the number and size of the largest LN involved were abstracted from the pathology report. For the tumor bed, the two dimensions were abstracted from the gross description. If they were not mentioned, they were measured on the glass slide. Then, the percentage of tumor cells and DCIS were estimated on a scale of 0 to 100. The RCB score and class were calculated as previously described. The tumor was considered pCR when there was no residual invasive carcinoma or positive LN (axillary or non-axillary) (ypTis/ypN0 or ypT0/ypN0). The RCB class was categorized on a scale from 0 (pCR) to III.^19^ The tumor was pathologically staged according to the guidelines of the American Joint Committee on Cancer (AJCC).^20^

### Clinical variables including immune-related adverse events

Clinical variables, such as age, race, menopausal status, tumor stage, nodal status, c-AJCC stage, and type of surgery (mastectomy or wide local excision; sentinel lymph node [SLN] or axillary lymph node dissection [ALND]), were collected from electronic medical records. In addition, systemic treatment, including name (CT or ICI) and number of doses, was also collected. Any irAE attributed to treatment up to the date of the last follow-up was also collected. Medical oncologists obtained information on irAE from retrospective reviews of the chart. Any grade irAE listed in the medical chart during CT + ICI or after completion of treatment was collected.

### Matching patients (control group that received CT alone)

The following matching criteria were applied: clinical (c-) AJCC stage (1, 2, 3), age (10-year increment), race (White, Black, other), and histological type (MpBC vs non-MpBC). Each institution provided cases treated with the CT + ICI regimen and potential control cases for matching (treated with CT alone). In the first round of matching, the CT + ICI-treated cases of each institution were matched with their own CT-treated control cases (*n* = 94). In the second round of matching, the left-over control cases were pooled and used as a resource to match more cases. In this round, 20 CT + ICI combination cases were matched. In the third round of matching, when a case did not have a perfect match, the criteria were relaxed around age and race. All cases had perfect matches except for 17. When the age did not match (10-year increment), the patient with the closest age was chosen. The cases that did not have a perfect match were 5 for race, 9 for age, and 3 for combined age and race. There were 5 patients with an age difference of more than 10 years (Table S1).

### Ethics approval and consent to participate

The institutional review board granted approval for the study from every participating institution (BDR # 055014 at RPCCC). The study was exempt from acquiring patients’ consents given minimal risk per the IRB.

### Statistical analysis

The clinical and pathological variables were summarized by group (CT and ICI+CT), pCR status, or irAE status using mean and SD for continuous variables and frequencies and relative frequencies for categorical variables. Comparisons were made using the Mann–Whitney *U* and Fisher’s exact tests, as appropriate. In the control group (CT alone), non-pCR status was modeled as a function of the pretreatment variables using a multivariable logistic regression model. The variables retained in the model were obtained by backwards selection (alpha exit = 0.3), and the final model was fit using Firth’s method. The performance of the model was evaluated using the associated ROC curve and the corresponding area under the curve (AUC). Based on the fitted model, the chance of non-pCR was calculated for all patients, and patients were categorized into quartiles based on the predicted risk of non-pCR (Q1 represents a low risk of non-pCR, Q4 represents a high risk of non-pCR, and Q2 and Q3 represent intermediate levels of risk). Within each quartile, the rates of non-pCR (and conversely pCR) between groups (CT vs ICI + CT) were compared using Fisher’s exact test. This information was used to identify subsets of patients in which the addition of ICI could (1) increase response or (2) not have any benefit. All analyses were performed in SAS v9.4 (Cary, NC) at a 2-sided significance level of .05.

## Results

The distribution of clinical and pathological variables for the CT + ICI and CT groups is shown in Table S2. The backbone of CT in the CT + ICI group was carboplatin in all patients except one. There was a perfect match between the two groups for c-AJCC stage and histological type (MpBC vs non-MpBC), a very close match for age, and a relatively good match for race. Pathologic complete response was achieved in 64 (50%) patients treated with CT + ICI vs 46 (35.9%) treated with CT alone (*P* = .023). The overall RCB score was lower in patients treated with a CT + ICI vs CT alone with a mean [SD] of 1.1 [1.4] vs 1.6 [1.5], respectively (*P* = .023) (Table S2).

Table 1 illustrates the clinical and pathological variables associated with pCR and non-pCR in both groups. The following variables were associated with pCR in both groups in univariable analyses: histologic type (IC-NST), NG-3, and high TILs. Unlike the CT alone group, a younger age of 48.2 years (± 12.3) vs 53.1 (+/- 12.7) and a lower c-N-stage were associated with pCR in the CT + ICI group. We also investigated the impact of the number of doses of CT + ICI on the rate of pCR. If ICI + CT arm, carboplatin and paclitaxel could be given every 3 weeks or weekly. To standardize, if paclitaxel or carboplatin was given every 3 weeks, we converted this to 3 weekly doses. If given weekly, carboplatin and paclitaxel were given x 12 doses, followed by doxorubicin and cyclophosphamide every 3 weeks along with pembrolizumab every 3 weeks, with any administration of ICI/type of chemotherapy considered as one dose. There were no differences in the mean number of CT and ICI according to pCR status (Table 1).

**Table 1.** Univariate analysis for clinical and pathological variables association with pCR and non-pCR in both groups.

		Cases (CT + ICI)			Control (CT alone)		
		**Non-pCR** 64 (50)**^a^**	**pCR** 64 (50)	*P* value	**Non-pCR** 82 (64.1)	**pCR** 46 (35.9)	*P* value
**Clinical**							
**Age at diagnosis**	**Mean [SD]**	53.1 [12.7]	48.2 [12.3]	.03	51.9 [12.2]	50.5 [12]	.6
**Race**	**White**	47 (73.4)	44 (68.8)	.38	68 (82.9)	34 (73.9)	.06
	**Black**	5 (7.8)	10 (15.6)		3 (3.7)	7 (15.2)	
	**Other**	12 (18.8)	10 (15.6)		11 (13.4)	5 (10.9)	
**c-T-Stage**	**1**	7 (10.9)	6 (9.4)	.1	6 (7.3)	4 (8.7)	.9
	**2**	32 (50)	45 (70.3)		46 (56.1)	28 (60.9)	
	**3**	18 (28.1)	10 (15.6)		24 (29.3)	11 (23.9)	
	**4**	7 (10.9)	3 (4.7)		6 (7.3)	3 (6.5)	
**c-N-Stage**	**0**	26 (40.6)	38 (59.4)	.034	39 (47.6)	22 (47.8)	.37
	**1**	22 (34.4)	8 (12.5)		23 (28)	8 (17.4)	
	**2**	12 (18.8)	16 (25.0)		14 (17.1)	13 (28.3)	
	**3**	4 (6.3)	2 (3.1)		6 (7.3)	3 (6.5)	
**c-AJCC stage**	**1**	1 (1.6)	2 (3.1)	.1	1 (1.2)	2 (4.3)	.28
	**2**	30 (46.9)	41 (64.1)		49 (59.8)	22 (47.8)	
	**3**	33 (51.6)	21 (32.8)		32 (39)	22 (47.8)	
**Type of surgery**	**Mastectomy**	38 (59.4)	33 (51.6)	.37	48 (58.5)	16 (34.8)	.008
	**Lumpectomy**	26 (40.6)	31 (48.4)		31 (37.8)	30 (65.2)	
**LN procedure**	**SLN**	35 (54.7)	60 (93.8)	<.001	27 (32.9)	28 (60.9)	.002
	**ALND**	29 (45.3)	4 (6.3)		55 (67.1)	18 (39.1)	
**ICI doses (*N*)** ^b^	**Mean [SD]**	7.4 [1.7]	7 [2.1]	.4	NA	NA	NA
**Paclitaxel** ^b^	**Mean [SD]**	10.7 [2.9]	11.4 [1.8]	.7	NA	NA	NA
**Carboplatin** ^b^	**Mean [SD]**	10 [3.2]	9.8 [3.4]	.7	NA	NA	NA
**Doxorubicin** ^b^	**Mean [SD]**	3.5 [1.2]	3.5 [1.3]	.64	NA	NA	NA
**Cyclophosphamide** ^b^	**Mean [SD]**	3.6 [1.3]	3.5 [1.3]	.52	NA	NA	NA
**Pathology of core needle biopsy**							
**Histologic type**	**IC-NST**	50 (78.1)	60 (93.8)	.013	70 (85.4)	46 (100)	.024
	**Apocrine**	7 (10.9)	0(0)		1 (1.2)	0 (0)	
	**Metaplastic**	7 (10.9)	4 (6.3)		11 (13.4)	0 (0)	
**Nottingham grade**	**2**	12 (18.8)	2 (3.1)	.005	16 (19.5)	3 (6.5)	.047
	**3**	52 (81.3)	62 (96.9)		66 (80.5)	43 (93.5)	
**TILs**	**Mean [SD]**	19.5 [23]	46.4 [27.9]	<.001	21.6 [19.2]	40.8 [25.1]	<.001
**Degree of necrosis**	**0**	0	29 (45.3)	.57	40 (48.8)	24 (52.2)	.97
	**1**	1	11 (17.2)		10 (12.2)	6 (13)	
	**2**	2	6 (9.4)		6 (7.3)	4 (8.7)	
	**3**	3	6 (9.4)		12 (14.6)	6 (13)	
	**4**	4	12 (18.8)		14 (17.1)	6 (13)	

Abbreviations: ALND, axillary lymph node dissection; IC-NST, invasive carcinoma of no special type; LN, lymph nodes; NA, not applicable; SLN, sentinel lymph node; TILs, tumor-infiltrating lymphocytes.

a *N* (%).

b ICI: Each administration prior to mastectomy/lumpectomy is considered as one dose.

Chemotherapy: Each administration is considered one dose; for paclitaxel and carboplatin—weekly administration was reported. If patients received paclitaxel or carboplatin Q3 week doses—these were converted to weekly doses. Doxorubicin and cyclophosphamide could be given every 2 or every 3 weeks.

### Variables associated with the benefit of CT + ICI and those benefiting from CT alone

**Figure 1.**
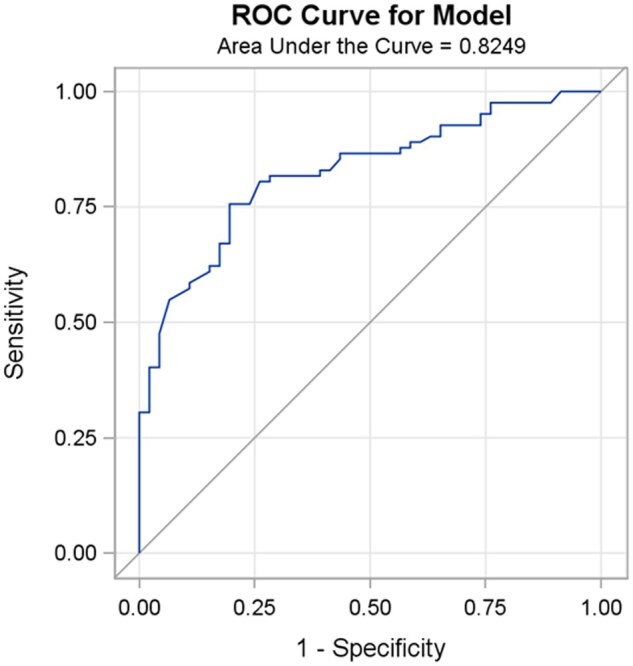
Receiver operating characteristic (ROC) curve for the multivariable logistic regression model predicting non-pCR in the control group (CT alone). The area under the curve (AUC) is 0.8249, indicating moderate predictive performance.

**Figure 2.**
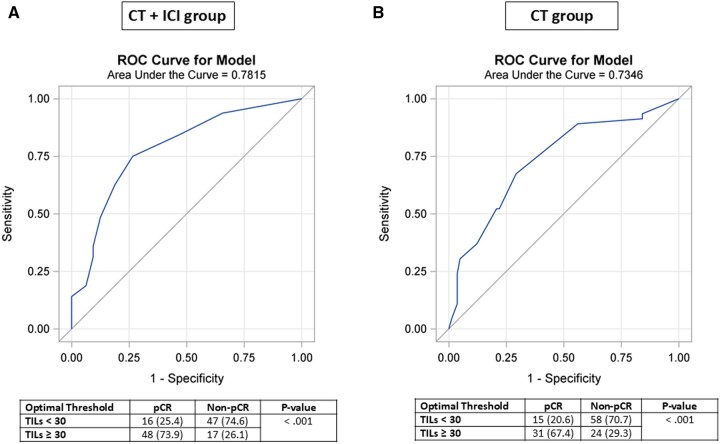
Receiver operating characteristic (ROC) curve based on TILs in both the CT + ICI group (A) and CT group (B). The optimal cutoff is identified using the Youden’s index criterion and the pCR rates are compared to above/below the cutoff using Fisher’s exact test. Note that the best cutoff for both groups is 30%.

To identify variables associated with benefit from CT + ICI, we first aimed to define variables associated with non-pCR in the CT (control) group. In the CT alone group, non-pCR was modeled as a function of pathological and clinical factors prior to treatment. From the final model, an ROC curve and the corresponding AUC were obtained. We found that the risk of non-pCR was mainly driven by low TILs, non-Black race, histological type (MpBC), and degree of necrosis (Table 2). The AUC was 82.49, indicating that these factors were reasonably predictive of the lack of response (Figure 1). Then, from this model, we generated an estimated risk of non-pCR, which was applied to the CT + ICI and CT groups. The predicted risk of non-pCR was categorized into four quartiles (Table 2). Next, we examined whether pCR rates differed between the control and CT + ICI groups within each of the risk groups. pCR rates were compared using Fisher’s exact test. We found that the CT + ICI group has a significantly higher pCR rate in the fourth risk quartile (control group with a high rate of non-pCR) (Table 3). Table 4 shows the clinical and pathological characteristics that were associated with benefiting from the CT + ICI group, that is, those in Q4 vs Q1 + 2 + 3: non-Black race (96.9% vs 88%, *P* = .04), MpBC histology (29.7% vs 1.6%, *P* < .01), lymph node-positive disease (54.6% vs 50%, *P* < .01), NG 2 (32.8% vs 6.3%, *P* < .01), with a lower mean TILS (12.8 ± 15 vs 36.7 ± 26.5, *P* < .01), which have a low likelihood of pCR with CT alone. The best cutoff for TILs was 30% for the CT + ICI and CT alone groups with AUC of 78.15 and 73.46, respectively (*P* < .001 for both) (Figure 2).

**Table 2.** Multivariable logistic regression analysis to predict non-pCR in control group (CT alone).

Variable	Levels	OR (95% CI)	*P*-value
**TILs**	**Unit increase**	0.97 (0.95, 0.99)	.001
**Race**	**Black vs White**	0.23 (0.05, 1.12)	.17
	**Other vs White**	0.62 (0.16, 2.37)	
**Nottingham grade**	**3 vs 2**	0.33 (0.08, 1.32)	.12
**Histology**	**MpBC vs Non-MpBC**	11.93 (0.59, 239.69)	.11
**c-N-stage**	**cN1+ vs cN0**	2.84 (0.78, 10.30)	.11
**c-AJCC stage**	**2 vs 1**	1.89 (0.12, 29.64)	.22
	**3 vs 1**	0.62 (0.03, 12.68)	
**Degree of necrosis**	**1/2 vs 0**	1.46 (0.48, 4.47)	.16
	**3/4 vs 0**	2.80 (0.98, 7.97)	

**Table 3.** pCR rate CT alone and CT + ICI cohorts within each of the risk groups.

Chance of non-pCR	pCR rate		*P* value
	CT alone	CT + ICI	
**First quartile**	18/27 (66.7)^a^	29/37 (78.4)	.39
**Second quartile**	20/38 (52.6)	16/26 (61.5)	.61
**Third quartile**	7/29 (24.1)	13/35 (37.1)	.29
**Fourth quartile**	1/34 (2.9)	6/30 (20)	.044

a *N* (%).

**Table 4.** Clinical and pathological variables association with low vs high non-pCR groups; identifying the specifications of patients who are at high risk of not achieving pCR by CT alone and could benefit from adding IT.

Overall		**Low non-pCR risk 192 (75)** ^a^	High non-pCR risk (fourth quartile) 64 (25)
**Clinical**			
**Age at diagnosis**	**Mean [SD]**	51.4 [12.5]	49.9 [11.9]
**Race**	**White**	145 (75.5)	48 (75)
	**Black**	23 (12)	2 (3.1)
	**Other**	24 (12.5)	14 (21.9)
**c-T-stage**	**1**	15 (7.8)	8 (12.5)
	**2**	115 (59.9)	36 (56.3)
	**3**	51 (26.6)	12 (18.8)
	**4**	11 (5.7)	8 (12.5)
**c-N-stage**	**0**	96 (50)	29 (45.3)
	**1**	35 (18.2)	26 (40.6)
	**2**	48 (25)	7 (10.9)
	**3**	13 (6.8)	2 (3.1)
**c-AJCC stage**	**1**	6 (3.1)	0 (0)
	**2**	96 (50)	46 (71.9)
	**3**	90 (46.9)	18 (28.1)
**Pathology of core needle biopsy**			
**Histologic type**	**IC-NST**	183 (95.3)	43 (67.2)
	**Apocrine**	6 (3.1)	2 (3.1)
	**Metaplastic**	3 (1.6)	19 (29.7)
**Nottingham grade**	**2**	12 (6.3)	21 (32.8)
	**3**	180 (93.8)	43 (67.2)
**TILs**	**Mean [SD]**	36.7 [26.5]	12.8 [15]
**Degree of necrosis**	**0**	88 (45.8)	26 (40.6)
	**1**	28 (14.6)	10 (15.6)
	**2**	23 (12)	3 (4.7)
	**3**	23 (12)	10 (15.6)
	**4**	30 (15.6)	15 (23.4)

Abbreviations: IC-NST, invasive carcinoma of no special type; TILs, tumor-infiltrating lymphocytes.

a *N* (%).

We also examined the data differently by defining the variables associated with benefiting from CT alone. To identify the clinical and pathological characteristics of patients who would have a high chance of achieving pCR with CT alone, we reversed the previously mentioned model, which has a similar AUC. The predicted chance of CT-induced pCR alone was categorized into five levels, with level 1 to 5, 5 being the highest. The frequency of the cases was 25 (19.5%), 27 (20.1%), 21 (16.4%), 30 (23.4%), and 25 (19.5%), respectively. The pCR rate in these groups increased from 0 (0%), to 5 (18.5%), to 6 (28.6%), to 18 (60%), and to 17 (68%), respectively. The chance of pCR for groups 4 and 5 was significantly higher compared to groups 1, 2, and 3 (*P* = .01). As expected, the clinical and pathological variables associated with benefit to CT alone included Black race, histological subtype of IC-NST, NG-3, and high TILs. However, c-AJCC stage had borderline significance (Table S3).

### Risk of immune-related adverse events for patients treated with CT + ICI

Any grade of immune-related adverse events (irAEs) was identified in 41 (32%) of the patients treated with CT + ICI. Patients who developed irAEs were more likely to achieve pCR than those who did not, 26 (63.4%) vs 15 (36.6%) (*P* = .037). Similarly, patients who developed irAE had a lower RCB score than those who did not with mean and [SD] 0.8 [1.3] vs 1.3 [1.4] (*P* = .03). There was a statistically significant association of irAE with pCR (OR 3.34, 95% CI 1.17-9.58) in multivariate adjustment while controlling for age, clinical AJCC stage, NG, race, TILs, and number of pembrolizumab (ICI) doses (Table 5). There was no statistically significant association between irAE and any of the chemotherapy agents, the number of pembrolizumab doses, or TILs (Table S4). A list of irAE experienced by patients during or after completion of treatment in the CT + ICI group is shown in Table S5.

**Table 5.** Association of variables with pathological complete response showing that occurrence of any grade irAE is associated with higher probability of pCR.

Variable	Level	OR (95% CI)	*P* value
**irAE**	**Yes vs No**	3.34 (1.17, 9.58)	.025
**Age**	**40-50 vs < 40**	0.64 (0.16, 2.54)	.30
	**50-60 vs < 40**	0.25 (0.06, 1.06)	
	**60-70 vs < 40**	0.41 (0.10, 1.74)	
	**≥70 vs < 40**	0.19 (0.02, 1.76)	
**c-AJCC stage**	**2 vs 1**	2.92 (0.09, 90.4)	.31
	**3 vs 1**	1.43 (0.05, 44.40)	
**Nottingham grade**	**3 vs 2**	11.05 (1.59, 76.86)	.015
**Race**	**Black vs White**	2.78 (0.56, 13.96)	.22
	**Other vs White**	0.47 (1.13, 1.74)	
**TILS**	**10-20 vs ≤10**	1.63 (0.37, 7.09)	<.001
	**20-50 vs ≤10**	8.27 (2.45, 27.90)	
	**>50 vs ≤10**	14.23 (3.44, 58.95)	
**ICI doses (*N*)**	**8+ vs < 8**	0.41 (0.14, 1.21)	.11

## Discussion

To our knowledge, this is the first multicenter study to compare the response rate of CT + ICI vs CT alone in the real world through a close matching process among patients with early-stage TNBC. Given the relatively recent approval of CT +ICI for early-stage TNBC, an academic collaboration among 4 institutions was performed for a robust analysis. Furthermore, we wanted to have minimal variation in histological interpretation between pathologists; therefore, the collaborators were chosen based on their prior experience (minimum of 10 years of experience). Similar to the results from the KN-522 trial, we found that patients treated with the CT + ICI combination had a significantly higher pCR rate compared to those treated with CT alone: 50% vs 35.9%, although the overall pCR rates were lower than those reported in the trial,^5^ which is reflective of experience with other real-world datasets where efficacy is always lower in real-world studies compared to clinical trials (using more stringent inclusion and exclusion criteria). It should be noted that the backbone of CT in this group was carboplatin in all patients except one. Furthermore, as expected, overall RCB scores were lower in patients treated with the CT + ICI combination compared to those treated with CT alone.^6^ More importantly, we found that there were potential variables associated with preferential benefit of addition of ICI to CT, including non-Black race, c-AJCC stage 2, metaplastic histology, NG-2, and low TILs. In contrast, we found variables that are associated with benefit from CT alone, include Black race, histological type of IC-NST, NG-3, and high TILs, where potentially use of ICI could be spared.

To date, only a few published studies have evaluated the real-world pCR to the CT + ICI regimen in early-stage TNBC. The pCR rate with ICI + CT varied among different studies from 34.9% to 63.6%.^21-24^ We have the largest group of patients who were treated with CT + ICI, with a pCR rate of 50%. There are many possible reasons for the observed variation in pCR rates among these studies. LeVee et al. found that patients of younger age and those who received 8 doses or more of pembrolizumab had a higher pCR rate. We found the following variables preferentially associated with pCR in the CT + ICI groups, including the histological type, NG-2, and low TILs (Table S6), compared to the CT group alone. The number of doses did not have an effect on the pCR rate. Therefore, the variation in the pCR between these studies could be explained in part by the selected cohort of patients who might have favorable clinical characteristics.

Furthermore, we calculated the RCB following published studies.^19^ We found a statistically significant difference in RCB scores between the CT + ICI and the CT alone group. Patients treated with CT + ICI had lower RCB scores than those in the CT alone group, which is similar to the findings reported in the KN-522 trial.^6^ Therefore, the benefit of CT + ICI is not only in achieving pCR but also in reducing RCB. We used pCR as the end point since it is a valid surrogate variable associated with better survival outcomes. Since our study, like the KN-522 trial, found a benefit in decreasing RCB, it would be important to follow up on these patients and evaluate for their survival (recurrence free and disease-specific survival). Given the recent approval of CT + ICI in the neoadjuvant setting for TNBC, there is not enough follow-up yet to report on the survival data.

We intended to define clinicopathological variables to identify certain patients who may benefit from CT alone or those who benefit from the addition of ICI. We collected a relatively large number of patients (*n* = 128) who were treated with CT + ICI and matched them with patients treated with CT alone. There was a perfect match for the c-AJCC stage and the histological type and a very close match for age and race. Furthermore, the percentage of TILs and NG was very close between the two cohorts, although they were not part of the matching process. When we divided the cases into four quartiles according to the predicted pCR rate, they were similarly distributed within each of the risk categories (*P* = .43). Therefore, we believe that our study is well designed to evaluate our objective. We found that non-Black patients, c-AJCC stage 2, histological type of MpBC, NG-2, and low TILs benefitted from the addition of ICI to CT vs CT alone. On the other hand, patients with Black race, histological type of IC-NST, NG-3, and high TILs have a high likelihood of achieving pCR with CT alone and could be spared an ICI. In quartile 4, which includes patients with non-Black race, metaplastic histology, lymph node-positive disease, NG 2, with lower mean TILS, the pCR rate with CT alone is 2.9% vs 20% with ICI + CT. This implies that for these patients, there is a >15% higher probability of pCR with the addition of ICI. This is a clinically relevant difference. It is worth noting that, unlike KN-522 clinical trial, we included c-AJCC stage 1 (*n* = 3) cases since the study is conducted in the real world. Moreover, apocrine type was not matched, but when we conducted a sensitivity analysis, the results remained consistent, underscoring the robustness of our findings.

The degree of TILs is currently established as a factor associated with pCR in patients with TNBC treated with NACT.^25^ It has also recently been found to associate with pCR in patients treated with KN-522 regimen.^21^ Furthermore, Leon-Ferre et al. found that early-stage TNBC with high TILs (>50%) that were not treated with adjuvant or neoadjuvant CT had better survival.^26^ We found that tumors with high TILs may achieve a similar pCR rate whether the patients were treated with ICI +CT or CT alone. A few studies found an association between TILs and response to IT.^27-29^ We found that tumors with low TILs may benefit from CT + ICI more than CT alone, which means that ICI, unlike CT alone, may be able to result in pCR even with low level of TILs in TNBC. In the KN-522 trial, pCR was consistent across different subgroups of PD-L1 expression. Loi et al. evaluated the role of TILs in patients treated with pembrolizumab vs chemotherapy for previously treated metastatic TNBC (KN-119 trial) and found that stromal TILs as low as 5% or more predicted response to pembrolizumab monotherapy.^30^ Several other studies as well as recent study from our group showed that a high tumor inflammation score was associated with pCR both with CT alone and with CT + ICI combination.^31^^,^^32^ Therefore, patients could potentially be triaged according to the level of TILs; patients whose tumor have high TILs (≥30%) receive CT alone and those with lower TILs (<30%) receive CT + ICI. However, since studies have shown that not only does the rate of TILs play a role in ICI response/resistance but also that the phenotypes of the cells, activation state, and spatial location are closely related,^33^ an additional study is warranted that incorporates clinical and pathological factors with a more in-depth analysis of TILs.

We found that non-Black patients benefit from CT + ICI. It may be due to racial differences in immune responses to tumors. Yao et al. found a stronger CD4 and B-cell response in black patients and a more exhausted CD8 T-cell profile.^34^ We also found that Black patients achieve pCR with CT alone at a high rate, which is the opposite of what has been published.^7^^,^^35^ It is possibly due to the small sample size in this group (*n* = 10). A study with a much larger cohort is needed to evaluate the interaction between race, TILs (level and function of various components), and the response to these two regimens.

In our study, we observed that there could be variability among the centers in terms of frequency of administration of carboplatin and paclitaxel—weekly or every 3 weeks as part of KEYNOTE 522 regimen. To standardize and enable robust analysis with sufficient power, we uniformly analyzed the doses, such that every 3 weeks carboplatin or paclitaxel was converted to weekly doses for analysis. In addition, doxorubicin and cyclophosphamide on KEYNOTE 522 could be given every 2 or every 3 weeks. Although there was no difference in pCR by the number of CT or ICI doses administered, a study limitation is the inability to examine the impact of CT dose reductions on pCR as the data on chemotherapy dose reductions or use of growth factor support was not collected. However, in another multi-center study across 4 centers in patients with early-stage TNBC treated with neoadjuvant chemo-immunotherapy led by our group, chemotherapy dose reductions or discontinuations (measured by relative dose intensity) were associated with a lower likelihood of pCR.^36^ These findings further highlight the need for biomarkers to judiciously select patients for ICI + CT, as CT dose reductions due to toxicities (irAEs, etc.) could potentially compromise efficacy.

Metaplastic breast carcinoma has the worst clinical outcomes among all subtypes of TNBC.^37^^,^^38^ They do not respond well to adjuvant or neoadjuvant therapies.^39-43^ Only case reports or studies with a small number of cases were published investigating the response rate of MpBC to ICIs in the neoadjuvant setting,^44^^,^^45^ or adjuvant setting.^46^ Abuhadra et al. prospectively evaluated the pCR rate for patients with MpBC treated with KN522; the pCR rate was 27% (4 of 15 patients).^45^ Data on the histologic subtypes included in the KN-522 trial are not available. We found that 4 of 11 MpBC achieved pCR in the CT + ICI group, while all 11 cases in the CT alone group did not. Therefore, we believe that the ICI regimen has an advantage in treating these tumors, which otherwise have poor clinical outcomes. In our study, we had 7 cases of apocrine differentiation in the ICI group, none of which achieved pCR, but we did not consider this subtype for matching. The number of cases in each of these histological subtypes is relatively small. Therefore, a larger study with more cases of these subtypes is needed to examine our results.

We observed that TILs, race, histology, lymph node positivity, and Nottingham grade are key clinicopathologic variables that may identify subgroup of patients with preferential benefit to ICI. Future randomized studies are needed to prospectively evaluate our findings from this study.

Finally, irAEs of any degree were found in 32% of the patients, most of whom were high-grade. The rate of serious irAEs was reported in 32.5% of patients in the KN-522 trial,^5^ consistent with our findings. We also found that patients who developed irAEs were three times more likely to achieve pCR than those who did not. This type of association was not mentioned in the KN-522 trial. An association between irAEs and improved outcomes of patients treated with ICIs has been reported in BC,^47^ melanoma,^48^ and lung cancer.^49^ However, all of these studies, including ours, differ in follow-up times and treatment exposures between patients who developed and did not develop irAEs. An additional limitation in our study is inability to grade the irAE or possibility of misclassification bias with irAEs, for example, diarrhea or rash as attribution to pembrolizumab or chemotherapy is difficult to discern from retrospective chart review. Multiple factors have been proposed to be associated with irAEs including TMB, germline mutations, and association of tumor-specific baseline neoantigens and CD8 positive T-cell infiltration.^50-52^ We encourage studies with a design similar to ours or in a clinical trial setting to evaluate these factors with TNBC.

## Conclusions

Our study has identified some clinicopathological characteristics in which ICI treatment should be prioritized including patients of non-Black race, tumors with c-AJCC stage 2, low TILs (<30%), NG-2, and MpBC. On the contrary, patients with tumors of IC-NST, NG-3, high TILs (≥30%) benefited with CT alone. Future studies with a larger number of patients are needed to validate our findings. Additionally, the survival analyses should also be conducted.

## Supplementary Material

oyaf231_Supplementary_Data

## Data Availability

The data are available upon reasonable request with the approval of the IRB from each collaborating institution.
