# Supplementary material for: Clinicopathological evaluation of triple-negative breast cancer treated with keynote-522 regimen
Source: Oncologist. 2025 Jul 24;30(9):oyaf231. doi: 10.1093/oncolo/oyaf231 (PMC12423027; doi:10.1093/oncolo/oyaf231)
Supplement: oyaf231_Supplementary_Data [file oyaf231_supplementary_data.zip › Supplemental tables revised - no track change.docx]

**Supplemental tables**

**Supplemental Table 1**: cases with no perfect matching (1 to 1) and how they are matched.

| **Case code** | **Variable not matched** | **Description of variable/s in chosen matches** |
| --- | --- | --- |
| **OHS-IT-76** | Race: AA | Race: CA |
| **UTSW-IT-28** | Age: 45 | Age: 51 |
| **UTSW-IT-19** | Age 46 | Age: 58 |
| **UTSW-IT-27** | Race: Hispanic | Race: CA |
| **UTSW-IT-31** | Race: Hispanic | Race: CA |
| **UCSD-IT-8** | Age: 50 | Age: 59 |
| **OHS-IT-66** | Age 30, race AA | Age 54, race CA |
| **UTSW-IT-16** | Age 25 | Age 38 |
| **OHS-IT-50** | Age 41, race other | Age 39, race CA |
| **UCSD-IT-16** | Age 25, race Hispanic | Age 52, race CA |
| **UTSW-IT-35** | Race AA | Race CA |
| **OHS-IT-71** | Race AA | Race CA |
| **UCSD-IT-5** | Age 29 | Age 41 |
| **UTSW-IT-34** | Age 53 | Age 50 |
| **OHS-IT-64** | Age 57 | Age 61 |
| **OHS-IT-78** | Age 57 | Age 61 |
| **UTSW-IT-38** | Age 59 | Age 62 |

**Supplemental Table 2**: Clinical and pathologic variables distribution for cases treated with CT +IT and CT alone.

|  |  | **Cases (CT+ICI)**  **(n=128)** | **Control (CT alone)**  **(n=128)** | **Overall**  **(n=256)** | **P Value** |
| --- | --- | --- | --- | --- | --- |
| **Clinical** | | | | | |
| **Age at Dx** | **Mean [SD]** | 50.7 [12.7] | 51.4 [12.1] | 51 [12.4] | .6 |
| **Race** | **White** | 91 (71.1) | 102 (79.7) | 193 (75.4) | .28 |
|  | **Black** | 15 (11.7) | 10 (7.8) | 25 (9.8) |  |
|  | **Other** | 22 (17.2) | 16 (12.5) | 38 (14.8) |  |
| **c-T-Stage** | **1** | 13 (10.2) | 10 (7.8) | 23 (9%) | .7 |
|  | **2** | 77 (60.2) | 74 (57.8) | 151 (59) |  |
|  | **3** | 28 (21.9) | 35 (27.3) | 63 (24.6) |  |
|  | **4** | 10 (7.8) | 9 (7.0%) | 19 (7.4) |  |
| **c-N-Stage** | **0** | 64 (50) | 61 (47.7) | 125 (48.8) | .87 |
|  | **1** | 30 (23.4) | 31 (24.2) | 61 (23.8) |  |
|  | **2** | 28 (21.9) | 27 (21.1) | 55 (21.5) |  |
|  | **3** | 6 (4.7) | 9 (7) | 15 (5.9) |  |
| **c-AJCC-Stage** | **1** | 3 (2.3) | 3 (2.3) | 6 (2.3) | 1 |
|  | **2** | 71 (55.5) | 71 (55.5) | 142 (55.5) |  |
|  | **3** | 54 (42.2) | 54 (42.2) | 108 (42.2) |  |
| **Type of Surgery** | **Mastectomy** | 71 (55.5) | 64 (50) | 135 (52) | .17 |
|  | **Lumpectomy** | 57 (44.5) | 61 (47.7) | 118 (46.1) |  |
|  | **Not Reported** | 0 (0) | 3 (2.3) | 3 (1.2) |  |
| **LN Procedure** | **SLN** | 95 (74.2) | 55 (43) | 150 (58) | <.001 |
|  | **ALND** | 33 (25.8) | 73 (57) | 106 (41.4) |  |
| **CNB** | | | | | |
| **Histologic Type** | **IC-NST** | 110 (85.9) | 116 (90.6) | 226 (88.3) | .1 |
|  | **Apocrine** | 7 (5.5) | 1 (0.8) | 8 (3.1) |  |
|  | **Metaplastic** | 11 (8.6) | 11 (8.6) | 22 (8.6) |  |
| **Nottingham Grade** | **2** | 14 (10.9) | 19 (14.8) | 33 (12.9) | .35 |
|  | **3** | 114 (89.1) | 109 (85.2) | 223 (87.1) |  |
| **TILs** | **Mean [SD]** | 33 [28.8] | 28.5 [23.3] | 30.7 [26.2] | .46 |
| **Degree of Necrosis** | **0** | 50 (39.1) | 64 (50) | 114 (44.5) | .3 |
|  | **1** | 22 (17.2) | 16 (12.5) | 38 (14.8) |  |
|  | **2** | 16 (12.5) | 10 (7.8) | 26 (10.2) |  |
|  | **3** | 15 (11.7) | 18 (14.1) | 33 (12.9) |  |
|  | **4** | 25 (19.5) | 20 (15.6) | 45 (17.6) |  |
| **Resection** | | | | | |
| **y-p-T-Stage** | **0** | 68 (53.1) | 57 (44.5) | 125 (48.8) | .008 |
|  | **1** | 43 (33.6) | 42 (32.8) | 85 (33.2) |  |
|  | **2** | 8 (6.3) | 25 (19.5) | 33 (12.9) |  |
|  | **3** | 5 (3.9) | 4 (3.1) | 9 (3.5) |  |
|  | **4** | 4 (3.1) | 0 (0) | 4 (1.6) |  |
| **Tumor size (mm)** | **Mean [SD]** | 9.4 [21.6] | 13.1 [21.6] | 11.3[21.7] | .038 |
| **y-p-N-Stage** | **0** | 96 (75) | 81 (63.3) | 177 (69.1) | .15 |
|  | **1** | 20 (15.6) | 34 (26.6) | 54 (21.1) |  |
|  | **2** | 6 (4.7) | 8 (6.3) | 14 (5.5) |  |
|  | **3** | 6 (4.7) | 5 (3.9) | 11 (4.3) |  |
| **y-p-AJCC-Stage** | **0** | 64 (50) | 48 (37.5) | 112 (43.8) | .03 |
|  | **1** | 30 (23.4) | 28 (21.9) | 58 (22.7) |  |
|  | **2** | 18 (14.1) | 37 (28.9) | 55 (21.5) |  |
|  | **3** | 16 (12.5) | 15 (11.7) | 31 (12.1) |  |
| **RCB Score** | **Mean [SD]** | 1.1 [1.4] | 1.6 [1.5] | 1.4 [1.4] | .023 |
| **RCB Class** | **0** | 64 (50) | 46 (35.9) | 110 (43) | .08 |
|  | **I** | 14 (10.9) | 20 (15.6) | 34 (13.3) |  |
|  | **II** | 37 (28.9) | 39 (30.5) | 76 (29.7) |  |
|  | **III** | 13 (10.2) | 23 (18.0) | 36 (14.1) |  |
| **pCR** | **non-pCR** | 64 (50) | 82 (64.1) | 146 (57) | .023 |
|  | **pCR** | 64 (50) | 46 (35.9) | 110 (43) |  |
| **Tumor/Tumor Bed Ratio** | **Mean [SD]** | 17.8 [29.9] | 25.7 [34.6] | 21.8 [32] | .05 |
| **LN-Total Resected (n)** | **Mean [SD]** | 7.1 [7.6] | 11.6 [8.9] | 9.3 [8.5] | <.001 |
| **LN-Positive (n)** | **Mean [SD]** | 1.1 [2.8] | 1.6 [4.7] | 1.3 [3.9] | .09 |
| **LN Size (mm)** | **Mean [SD]** | 2.1 [5.6] | 4.1 [9] | 3.1 [7.6] | .023 |

*N(%)

**Supplemental Table 3**: Clinical and Pathological Variables Association with Low vs. High pCR Groups; identifying the specifications of patients who are who have high probability of achieving pCR by CT alone and don’t benefit from adding IT.

|  |  | **Low pCR chance**  **73 (57)*** | **High pCR Chance**  **55 (43)** | **P value** |
| --- | --- | --- | --- | --- |
| **Clinical** | | | | |
| **Age at Diagnosis** | **Mean [SD]** | 50.6 [12] | 52.4 [12.1] | .4 |
| **Race** | **White** | 60 (82.2) | 42 (76.4) | .036 |
|  | **Black** | 2 (2.7) | 8 (14.5) |  |
|  | **Other** | 11 (15.1) | 5 (9.1) |  |
| **c-T-Stage** | **1** | 6 (8.2) | 4 (7.3) | .98 |
|  | **2** | 43 (58.9) | 31 (56.4) |  |
|  | **3** | 19 (26) | 16 (29.1) |  |
|  | **4** | 5 (6.8) | 4 (7.3) |  |
| **c-N-Stage** | **0** | 35 (47.9) | 26 (47.3) | .15 |
|  | **1** | 22 (30.1) | 9 (16.4) |  |
|  | **2** | 11 (15.1) | 16 (29.1) |  |
|  | **3** | 5 (6.8) | 4 (7.3) |  |
| **c-AJCC-Stage** | **1** | 1 (1.4) | 2 (3.6) | .06 |
|  | **2** | 47 (64.4) | 24 (43.6) |  |
|  | **3** | 25 (34.2) | 29 (52.7) |  |
| **Pathology on CNB** | | | | |
| **Histologic Type** | **IC-NST** | 61 (83.6) | 55 (100) | .007 |
|  | **Apocrine** | 1 (1.4) | 0 (0) |  |
|  | **Metaplastic** | 11 (15.1) | 0 (0) |  |
| **Nottingham Grade** | **2** | 17 (23.3) | 2 (3.6) | .002 |
|  | **3** | 56 (76.7) | 53 (96.4) |  |
| **TILs** | **Mean [SD]** | 15.9 [13.4] | 45.3 [23.2] | <.001 |
| **Degree of Necrosis** | **0** | 32 (43.8) | 32 (58.2) | 0.1 |
|  | **1** | 9 (12.3) | 7 (12.7) |  |
|  | **2** | 4 (5.5) | 6 (10.9) |  |
|  | **3** | 12 (16.4) | 6 (10.9) |  |
|  | **4** | 16 (21.9) | 4 (7.3) |  |

**Supplemental Table 4:** Clinical and pathological variables association with irAE vs. no irAE

|  |  | **No irAE**  **87 (68)** | **irAE**  **41 (32)** | **P value** |
| --- | --- | --- | --- | --- |
| **Clinical** | | | | |
| **Age at diagnosis** | **Mean [SD]** | 50.7 [12.6] | 50.7 [13.1] | .88 |
| **Race** | **White** | 61 (70.1) | 30 (73.2) | .51 |
|  | **Black** | 9 (10.3) | 6 (14.6) |  |
|  | **Other** | 17 (19.5) | 5 (12.2) |  |
| **c-T-Stage** | **1** | 9 (10.3) | 4 (9.8) | 1 |
|  | **2** | 52 (59.8) | 25 (61) |  |
|  | **3** | 19 (21.8) | 9 (22) |  |
|  | **4** | 7 (8) | 3 (7.3) |  |
| **c-N-Stage** | **0** | 43 (49.4) | 21 (51.2) | .63 |
|  | **1** | 23 (26.4) | 7 (17.1) |  |
|  | **2** | 17 (19.5) | 11 (26.8) |  |
|  | **3** | 4 (4.6) | 2 (4.9) |  |
| **c-AJCC-Stage** | **1** | 2 (2.3) | 1 (2.4) | .99 |
|  | **2** | 48 (55.2) | 23 (56.1) |  |
|  | **3** | 37 (42.5) | 17 (41.5) |  |
| **ICI Doses (N)** | **Mean [SD]** | 7.2 [1.8] | 7.1 [2.1] | .87 |
| **ICI Doses** | **< 8** | 25 (30.1) | 16 (40) | .28 |
|  | **8+** | 58 (69.9) | 24 (60) |  |
| **Paclitaxel** | **Mean [SD]** | 11.1 [2.6] | 11.1 [2] | .23 |
| **Carboplatin** | **Mean [SD]** | 9.9 [3.4] | 9.8 [3.2] | .54 |
| **Doxorubicin** | **Mean [SD]** | 3.5 [1.2] | 3.6 [1.2] | .79 |
| **Cyclophosphamide** | **Mean [SD]** | 3.5 [1.3] | 3.6 [1.2] | .91 |
| **CNB** | | | | |
| **Histologic Type** | **IC-NST** | 73 (83.9) | 37 (90.2) | .53 |
|  | **Apocrine** | 6 (6.9) | 1 (2.4) |  |
|  | **Metaplastic** | 8 (9.2) | 3 (7.3) |  |
| **Nottingham Grade** | **2** | 8 (9.2) | 6 (14.6) | .36 |
|  | **3** | 79 (90.8) | 35 (85.4) |  |
| **TILs** | **Mean [SD]** | 32.4 [29.8] | 34.1 [26.8] | .52 |
| **Degree of Necrosis** | **0** | 32 (36.8) | 18 (43.9) | .8 |
|  | **1** | 14 (16.1) | 8 (19.5) |  |
|  | **2** | 12 (13.8) | 4 (9.8) |  |
|  | **3** | 10 (11.5) | 5 (12.2) |  |
|  | **4** | 19 (21.8) | 6 (14.6) |  |
| **Resection** | | | | |
| **y-p-T-Stage** | **0** | 41 (47.1) | 27 (65.9) | .17 |
|  | **1** | 31 (35.6) | 12 (29.3) |  |
|  | **2** | 8 (9.2) | 0 (0) |  |
|  | **3** | 4 (4.6) | 1 (2.4) |  |
|  | **4** | 3 (3.4) | 1 (2.4) |  |
| **Tumor size (mm)** | **Mean [SD]** | 10 [18.2] | 8.1 [27.8] | .06 |
| **y-p-N-Stage** | **0** | 61 (70.1) | 35 (85.4) | .08 |
|  | **1** | 18 (20.7) | 2 (4.9) |  |
|  | **2** | 3 (3.4) | 3 (7.3) |  |
|  | **3** | 5 (5.7) | 1 (2.4) |  |
| **y-p-AJCC-Stage** | **0** | 38 (43.7) | 26 (63.4) | .1 |
|  | **1** | 21 (24.1) | 9 (22) |  |
|  | **2** | 16 (18.4) | 2 (4.9) |  |
|  | **3** | 12 (13.8) | 4 (9.8) |  |
| **RCB Score** | **Mean [SD]** | 1.3 [1.4] | 0.8 [1.3] | .03 |
| **RCB Class** | **0** | 38 (43.7) | 26 (63.4) | .22 |
|  | **I** | 11 (12.6) | 3 (7.3) |  |
|  | **II** | 28 (32.2) | 9 (22) |  |
|  | **III** | 10 (11.5) | 3 (7.3) |  |
| **pCR** | **non-pCR** | 49 (56.3) | 15 (36.6) | .037 |
|  | **pCR** | 38 (43.7) | 26 (63.4) |  |
| **Tumor/Tumor Bed Ratio** | **Mean [SD]** | 21.3 [32.6] | 10.4 [21.8] | .024 |
| **LN-Positive (n)** | **Mean [SD]** | 1.3 [3.2] | 0.6 [2] | .08 |
| **LN Size (mm)** | **Mean [SD]** | 2.2 [5.2] | 1.7 [6.4] | .09 |

**Supplemental Table 5:** List of Immune related adverse events

|  | **Case ID** | **Immune related adverse events** |
| --- | --- | --- |
| 1 | OHS-IT-11 | Transaminitis, fatigue |
| 2 | OHS-IT-14 | Adrenal insufficiency |
| 3 | OHS-IT-16 | Hypothyroidism, rash |
| 4 | OHS-IT-21 | Transaminitis, joint pain |
| 5 | OHS-IT-22 | Pneumonitis |
| 6 | OHS-IT-25 | Polymyalgia rheumatica |
| 7 | OHS-IT-32 | Hypothyroidism |
| 8 | OHS-IT-35 | Ocular sicca syndrome, adrenal insufficiency |
| 9 | OHS-IT-36 | Diarrhea |
| 10 | OHS-IT-37 | Transaminitis |
| 11 | OHS-IT-41 | Rash |
| 12 | OHS-IT-44 | Ocular sicca syndrome |
| 13 | OHS-IT-57 | Transaminitis |
| 14 | OHS-IT-62 | Mouth ulcer, rash |
| 15 | OHS-IT-66 | Hyperthyroidism, adrenal insufficiency |
| 16 | OHS-IT-71 | Myositis |
| 17 | OHS-IT-73 | Rash, fatigue, diarrhea |
| 18 | OHS-IT-75 | Pneumonitis |
| 19 | OHS-IT-76 | Rash, transaminitis |
| 20 | RPCI-IT-01 | Rash |
| 21 | RPCI-IT-05 | Thyrotoxicosis, adrenocortical insufficiency |
| 22 | RPCI-IT-06 | Pruritis, adrenocortical insufficiency, rash |
| 23 | RPCI-IT-07 | Diarrhea |
| 24 | RPCI-IT-11 | Diarrhea, pruritis |
| 25 | RPCI-IT-15 | Rash |
| 26 | RPCI-IT-16 | Pruritis |
| 27 | RPCI-IT-19 | Diarrhea, pruritis |
| 28 | RPCI-IT-21 | Hepatic failure |
| 29 | RPCI-IT-22 | Colitis |
| 30 | RPCI-IT-24 | Hypothyroidism |
| 31 | UCSD-IT-01 | Hypothyroidism |
| 32 | UCSD-IT-15 | Hypothyroidism |
| 33 | UCSD-IT-17 | Adrenal insufficiency |
| 34 | UTSW-IT-09 | Nephritis |
| 35 | UTSW-IT-12 | Myositis |
| 36 | UTSW-IT-13 | Adrenal insufficiency |
| 37 | UTSW-IT-18 | Hypothyroidism |
| 38 | UTSW-IT-28 | Pneumonitis |
| 39 | UTSW-IT-33 | Pneumonitis |
| 40 | UTSW-IT-37 | Hypothyroidism |
| 41 | UTSW-IT-43 | Adrenal insufficiency |

**Supplemental Table 6**: Multivariable analysis for clinical and pathological variables association with pCR and non-pCR in both groups

| **Variable** | **Unites** | **OR (95% CI)** | **P value** |
| --- | --- | --- | --- |
| **CT + ICI group** | | | |
| **Age** | 40-50 vs. < 40 | 0.34 (0.09, 1.38) | 0.25 |
|  | 50-60 vs. < 40 | 0.23 (0.06, 0.94) |  |
|  | 60-70 vs. < 40 | 0.28 (0.07, 1.17) |  |
|  | 70+ vs. < 40 | 0.14 (0.02, 1.19) |  |
| **c-N-Stage** | N1+ vs. N0 | 0.60 (0.25, 1.42) | 0.24 |
| **Histologic Type** | Apocrine vs. IC-NST | 0.07 (0.01, 1.69) | 0.047 |
|  | MpBC vs. IC-NST | 0.22 (0.05, 1.00) |  |
| **Nottingham Grade** | 3 vs. 2 | 6.53 (1.24, 34.50) | 0.027 |
| **TILs** | Per unit increase (10%) | 1.04 (1.02, 1.05) | < 0.001 |
| **CT alone group** | | | |
| **Histologic Type** | Apocrine vs. IC-NST | 0.43 (0.01, 69.58) | 0.34 |
|  | MpBC vs. IC-NST | 0.11 (0.01, 2.25) |  |
| **Nottingham Grade** | 3 vs. 2 | 2.22 (0.60, 8.23) | 0.23 |
| **TILs** | Per unit increase (10%) | 1.03 (1.01, 1.05) | 0.001 |
